# NF‐κB inhibition reverses acidic bile‐induced miR‐21, miR‐155, miR‐192, miR‐34a, miR‐375 and miR‐451a deregulations in human hypopharyngeal cells

**DOI:** 10.1111/jcmm.13591

**Published:** 2018-03-08

**Authors:** Sotirios G. Doukas, Dimitra P. Vageli, Clarence T. Sasaki

**Affiliations:** ^1^ The Yale Larynx laboratory Department of Surgery Yale School of Medicine New Haven CT USA

**Keywords:** BAY 11‐7082, bile acids, hypopharyngeal cancer, miR‐155, miR‐192, miR‐21, miR‐34a, miR‐375, miR‐451a, NF‐κB

## Abstract

We previously demonstrated that acidic bile activates NF‐κB, deregulating the expression of oncogenic miRNA markers, in pre‐malignant murine laryngopharyngeal mucosa. Here, we hypothesize that the in vitro exposure of human hypopharyngeal cells to acidic bile deregulates cancer‐related miRNA markers that can be reversed by BAY 11‐7082, a pharmacologic NF‐κB inhibitor. We repetitively exposed normal human hypopharyngeal primary cells and human hypopharyngeal keratinocytes to bile fluid (400 μmol/L), at pH 4.0 and 7.0, with/without BAY 11‐7082 (20 μmol/L). We centred our study on the transcriptional activation of oncogenic miR‐21, miR‐155, miR‐192, miR‐34a, miR‐375, miR‐451a and NF‐κB‐related genes, previously linked to acidic bile‐induced pre‐neoplastic events. Our novel findings in vitro are consistent with our hypothesis demonstrating that BAY 11‐7082 significantly reverses the acidic bile‐induced oncogenic miRNA phenotype, in normal hypopharyngeal cells. BAY 11‐7082 strongly inhibits the acidic bile‐induced up‐regulation of miR‐192 and down‐regulation of miR‐451a and significantly decreases the miR‐21/375 ratios, previously related to poor prognosis in hypopharyngeal cancer. This is the first in vitro report that NF‐κB inhibition reverses acidic bile‐induced miR‐21, miR‐155, miR‐192, miR‐34a, miR‐375 and miR‐451a deregulations in normal human hypopharyngeal cells, suggesting that acidic bile‐induced events are directly or indirectly dependent on NF‐κB signalling.

## 1 INTRODUCTION

Hypopharyngeal cancer is one of the most aggressive subtypes of head and neck squamous cell carcinoma (HNSCC).^1^ Recent data from cancer statistics reveal poor prognosis, even for early stages of the disease, with an overall 5‐year survival of 24%.^1^ Tobacco and chronic alcohol have long been considered carcinogenic risk factors of laryngopharyngeal and hypopharyngeal cancer.^2^, ^3^ Gastroduodenal reflux disease (GDRD), a variant of gastro‐oesophageal reflux disease (GERD), has also recently been considered a risk factor that may exert independent carcinogenic potential related to chronic effects of toxic gastroduodenal fluid (GDF) on hypopharyngeal epithelial cells.^4^, ^5^, ^6^, ^7^

Although, it has been found that gastroduodenal refluxate can extend to the upper aerodigestive tract of patients,^5^, ^6^, ^7^ the tumorigenic effect of GDF is not yet well understood. Recent in vitro and in vivo studies have defined NF‐κB to be a possible mechanistic link between acidic bile or GDF and early pre‐neoplastic events in laryngopharyngeal mucosa.^8^, ^9^, ^10^ Specifically, the combination of bile and acid (pH ≤4.0) constitutively activates NF‐κB, up‐regulating the expression of cancer‐related genes and deregulating the expression of oncogenic miRNA markers, such as “oncomirs” miR‐21, miR‐192, miR‐155 and “tumour suppressors” miR‐34a, miR‐375 and miR‐451a, in pre‐malignant lesions of murine laryngopharyngeal mucosa.^9^, ^10^ Here, we describe an in vitro model exploring repetitive exposures of normal human hypopharyngeal cells to acidic bile, with and without BAY 11‐7082, a pharmacologic inhibitor of NF‐κB.^11^ We hypothesize that NF‐κB inhibitor is capable of preventing the acidic bile‐induced up‐regulation of “oncomirs” miR‐21, miR‐155 and miR‐192 and down‐regulation of “tumour suppressor” miR‐34a, miR‐375 and miR‐451a, previously associated with laryngopharyngeal cancer,^12^, ^13^, ^14^, ^15^, ^16^, ^17^ providing insight into interactions of transcriptionally active NF‐κB with cancer‐related miRNA markers.

MicroRNA (miRNA) molecules have been considered to play an important role in both inflammation and cancer,^18^ modulating the expression of genes by causing target mRNA degradation or inhibiting their translation.^19^ Specifically, some miRNAs, such as “oncomirs” and “tumour suppressor” miRNAs, show altered expression levels in tumour cells compared to normal cells (up‐regulated or down‐regulated) and are capable of contributing to carcinogenesis, demonstrating a significant regulating role in the multistep process of cancer initiation and progression.^20^ Previous studies have demonstrated that deregulation of “oncomirs” miR‐21, miR‐155, miR‐192 and tumour suppressor miR‐375, miR‐451a and miR‐34a is associated with laryngopharyngeal cancer.^12^, ^13^, ^14^, ^15^, ^16^, ^17^ Moreover, an independent association has been demonstrated between NF‐κB activation and up‐regulation of oncogenic miR‐21 and/or down‐regulation of tumour suppressor miR‐34a and miR‐451a.^21^, ^22^, ^23^

BAY 11‐7082 was selected as a reliable inhibitor of NF‐κB pathway that has been widely used in many studies exploring the effect of NF‐κB.^11^, ^24^, ^25^ It has been suggested that BAY 11‐7082 offers the most rapid and potent antitumour effect among other NF‐κB inhibitors 24 and can possibly be used as a sensitizer of anticancer therapy,^26^, ^27^ increasing the intrinsic susceptibility of cancer cells to chemotherapeutic agents.^28^

Evidence that inhibition of acidic bile‐induced NF‐κB activation effectively reverses the altered cancer‐related miRNA phenotype will encourage the in vivo application of NF‐κB inhibitors, as possible preventers of acidic bile effect in hypopharyngeal mucosa.

## 2 MATERIALS AND METHODS

### 2.1 Cell culture

#### 2.1.1 In vitro exposure to acidic bile with and without BAY 11‐7082

##### Acidic bile treatment

We performed a repetitive exposure of human hypopharyngeal primary cells (HHPC) (2nd passage) (Celprogen Inc. CA, USA) and telomerase‐immortalized human hypopharyngeal keratinocytes (HHK) (4th passage) 8 to bile (pH 4.0 and pH 7.0) and corresponding controls (pH 4.0 and pH 7.0), for 10‐15 minutes, 3 times per day, for 5 days, as previously described.^8^ Bile fluid consisted of a mixture of conjugated bile salts (400 μmol/L) considered to be “physiologic” 29, 30 (Supplementary Methods).

Experimental groups included HHPC and HHK repetitively exposed to (a) acidic bile at pH 4.0, the cut‐off of reflux disease 31, 32 and (b) neutral bile, containing the same bile salts mixture, at pH 7.0. Control groups included (a) acid control (pH 4.0), and (b) neutral control (pH 7.0) with identical media used in experimental groups (Supplementary Methods).

##### Acidic bile + BAY‐11‐7082 treatment

In parallel with acidic bile treatment, we performed an additional procedure of repetitive exposure of HHPC (2nd passage) and HHK (4th passage) to acidic bile with BAY 11‐7082, a pharmacologic inhibitor of NF‐κB (Calbiochem © 2016 EMD Millipore Corporation; Germany),^11^ for 10‐15 minutes, 3 times per day, for 5 days. Experimental groups included an identical procedure of repetitive exposure of HHPC and HHK to bile at pH 4.0 and 7.0, as described above, in combination with 20 μmol/L of BAY 11‐7082. Control groups included a repetitive exposure to acid alone (pH 4.0) and neutral control (pH 7.0) in combination with 20 μmol/L of BAY 11‐7082, with identical media used in experimental groups (Supplementary Methods). We also used untreated cells as negative control‐ and DMSO‐treated groups, as reference control for the NF‐κB inhibitor vehicle. DMSO was used at concentrations similar to those used for BAY 11‐7082 solubilization.

At the end of treatment, media were removed and cells or cell extracts were analysed.

### 2.2 Luciferase assay

We performed a luciferase assay in order to monitor the transcriptional activity of the NF‐κB in HHPC exposed to acidic bile and corresponding controls, with or without the pharmacologic inhibitor of NF‐κB, BAY 11‐7082. We used Firefly luciferase Assay system (Promega Corporation, Madison, WI, USA), Lipofectamine^®^ 2000 (Invitrogen™) and pGL4.32[luc2P/NF‐κB‐RE/Hygro] Vector, encoded with the firefly luciferase reporter gene (luc2P) driven by five copies of an NF‐κB enhancer element during the first 48 hour in culture, and control vector (pGL4.27[luc2P/minP/Hygro]), and in accordance with the manufacturer's procedure. Equal number of cells was transfected with NF‐κB or control luciferase vector. We performed triplicate assays for each treatment condition (bile with or without NF‐κB inhibitor and corresponding controls, at pH 4.0 and pH 7.0). At the end of treatments, luminescence was measured using a luminometer (Infinite^®^ M1000 PRO, TECAN) and i‐control™ software. We expressed NF‐κB activity as ratios of mean values [values for NF‐κB reporter (luc2P/NF‐kB‐RE), against the mean value for control (luc2P)] calculated in treated HHPC for each condition. Finally, we expressed the alterations of NF‐κB activity induced by BAY 11‐7082 as ratios of relative NF‐κB activity (with/without NF‐κB inhibitor). (Data were obtained from three independent experiments).

### 2.3 miRNA analysis

We performed miRNA analysis in order to determine the expression levels of miR‐21, ‐155, ‐192, ‐34α, ‐375 and ‐451α, previously characterized in laryngopharyngeal cancer,^12^, ^13^, ^14^, ^15^, ^16^, ^17^ in normal human hypopharyngeal cells, HHPC and HHK, exposed to acidic bile (pH 4.0), neutral bile (pH 7.0), acid (pH 4.0) and neutral control (pH 7.0) fluids, with or without pharmacologic inhibitor BAY 11‐7082.^11^ We estimated relative expression levels (target miRNA/RNU6B) for each specific miRNA marker, in each experimental and control group treated with or without NF‐κB inhibitor (CFX96TM software; Bio‐Rad, CA, USA) (Supplementary Methods & Table S1). (Data were obtained from three independent experiments).

We used the same pool of total RNA to determine, by qPCR, the effect of BAY 11‐7082 on transcriptional levels of RELA(p65), TNF‐α, IL‐1β, IL‐6 and STAT3 in acidic bile‐treated and control HHPC with or without BAY 11‐7082, as previously described^33^ (Supplementary Methods & Table S2). These genes were selected because they demonstrated an increased transcriptional activity under acidic bile exposure of HHPC 8, 9 that was prevented by BAY 11‐7082 in our previous study.^33^

### 2.4 Immunofluorescence assay

We performed an immunofluorescence (IF) assay in HHPC, as previously described^8^, ^9^, ^10^ (Supplementary Methods) to explore the effect of NF‐κB inhibitor (20 μmol/L of BAY 11‐7082) on the acidic bile‐induced nuclear translocation of phospho‐NF‐κB (p65, Ser536) and phospho‐STAT3 (Tyr705), previously shown to be up‐regulated in 45‐day acidic bile‐treated murine laryngopharyngeal mucosa.^10^

### 2.5 Cell viability assay

We performed a cell viability assay, using CellTiter‐Glo^®^ Luminescent Cell Viability Assay (Promega) to monitor the effect of NF‐κB inhibitor, BAY 11‐7082, on viability of HHPC and HHK treated with bile at pH 4.0 and pH 7.0, and corresponding controls, as described in Supplementary Methods. We determined cell viability by comparing the mean values of cells exposed to NF‐κB inhibitor against the mean value of cells that were not exposed to inhibitor, for each experimental and control group. Statistically significant difference in cell viability was determined using paired test and *P* value <.05 (Graph Pad Prism 6.0).

### 2.6 Statistical analysis

We performed statistical analysis, using GraphPad Prism 6 software and one‐way ANOVA (by Friedman and Dunn's multiple analysis test; *P*‐values <.05) to compare expression changes of the analysed miRNA markers and the analysed genes induced by BAY 11‐7082 (with/without NF‐kB inhibitor) between different experimental and control groups. We also used *t* test analysis (multiple comparisons by Holm‐Sidak) to reveal differential expression (*P*‐values) for each analysed miRNA marker in treated cells, with and without NF‐κB inhibitor. Finally, we performed a *Pearson* correlation to estimate the correlation coefficient between BAY 11‐7082‐induced miRNA and mRNA expression levels, as well as between “oncomirs” and “tumour suppressor” miRNA levels, of different treated groups (*P*‐values < .05).

## 3 RESULTS

### 3.1 BAY 11‐7082 effectively reduces acidic bile‐induced NF‐κB transcriptional activity in normal human hypopharyngeal cells

**Figure 1:**
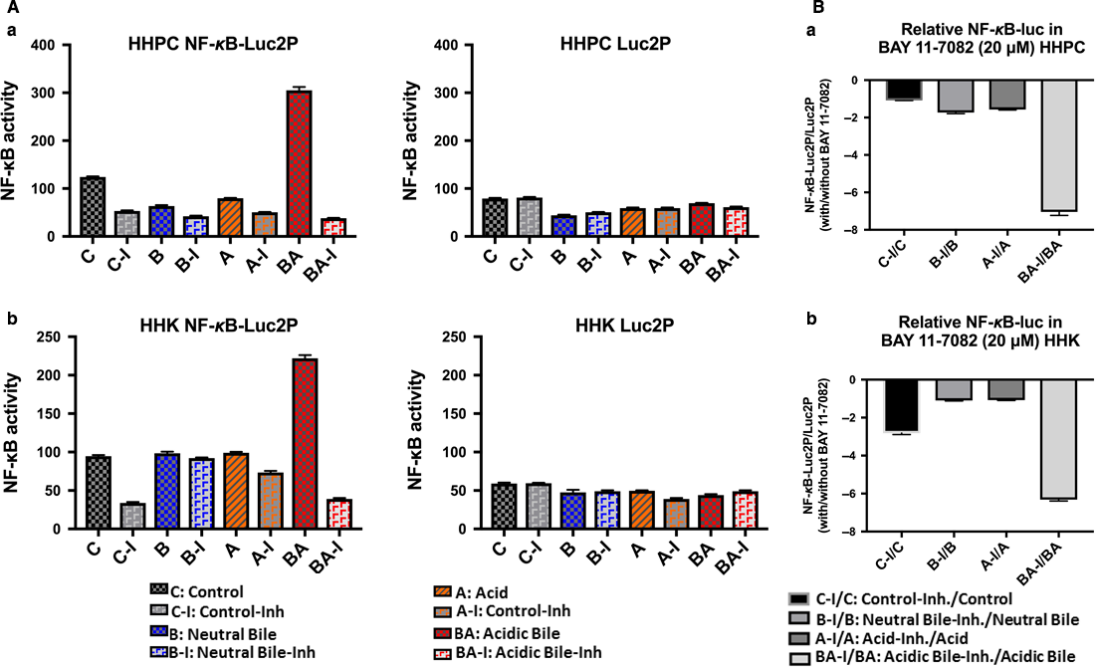
Luciferase assay demonstrates that 20 μmol/L of BAY 11‐7082 prevents the NF‐κB transcriptional activity in acidic bile‐treated normal human hypopharyngeal cells (HHPC) and human hypopharyngeal keratinocytes (HHK). A, Columns of graphs represent luciferase activity (mean ± standard error of three independent experiments) in (a) HHPC and (b) HHK, transfected with control luciferase reporter (luc2P) and NF‐κB luciferase responsive element (luc2P‐NF‐κB‐RE). B, Columns of graphs represent NF‐κB relative transcriptional activity (luc2P‐NF‐κB‐RE: NF‐κB luciferase responsive element/luc2P: control luciferase reporter) with/without BAY 11‐7082, in (a) HHPC and (b) HHK. Luc, luciferase

To investigate the effect of 20 μmol/L of BAY 11‐7082 in NF‐κB transcriptional activity of acidic bile‐treated normal human hypopharyngeal cells, HHPC and HHK, we used an NF‐κB luciferase assay (Figure 1), demonstrating that 20 μmol/L of pharmacologic inhibitor BAY 11‐7082 effectively prevented the transcriptional activity of NF‐κB in acidic bile‐treated HHPC and HHK. We observed that cells exposed to neutral bile, acid or neutral control with NF‐κB inhibitor also demonstrated a reduced transcriptional activity of NF‐κB, compared to those treated without BAY 11‐7082 (Figure 1A). However, the acidic bile‐treated group demonstrated the most reduced ratios of relative NF‐κB transcriptional activity (NF‐κB luciferase responsive element/control luciferase reporter) with/without BAY 11‐7082, relative to neutral bile, acid or neutral control groups (Figure 1B).

### 3.2 BAY 11‐7082 reverses the acidic bile‐induced deregulation of cancer‐related miRNAs in normal human hypopharyngeal cells

We performed miRNA analysis, by qPCR, in normal human hypopharyngeal cells, HHPC and HHK, exposed to acidic bile (pH 4.0), neutral bile (pH 7.0) and corresponding controls, with and without NF‐κB inhibitor (BAY 11‐7082). We analysed specific miRNA markers, previously characterized as “oncomirs”, such as miR‐21, miR‐155 and miR‐192, or “tumour suppressors”, such as miR‐34a, miR‐375 and miR‐451a.

**Figure 2:**
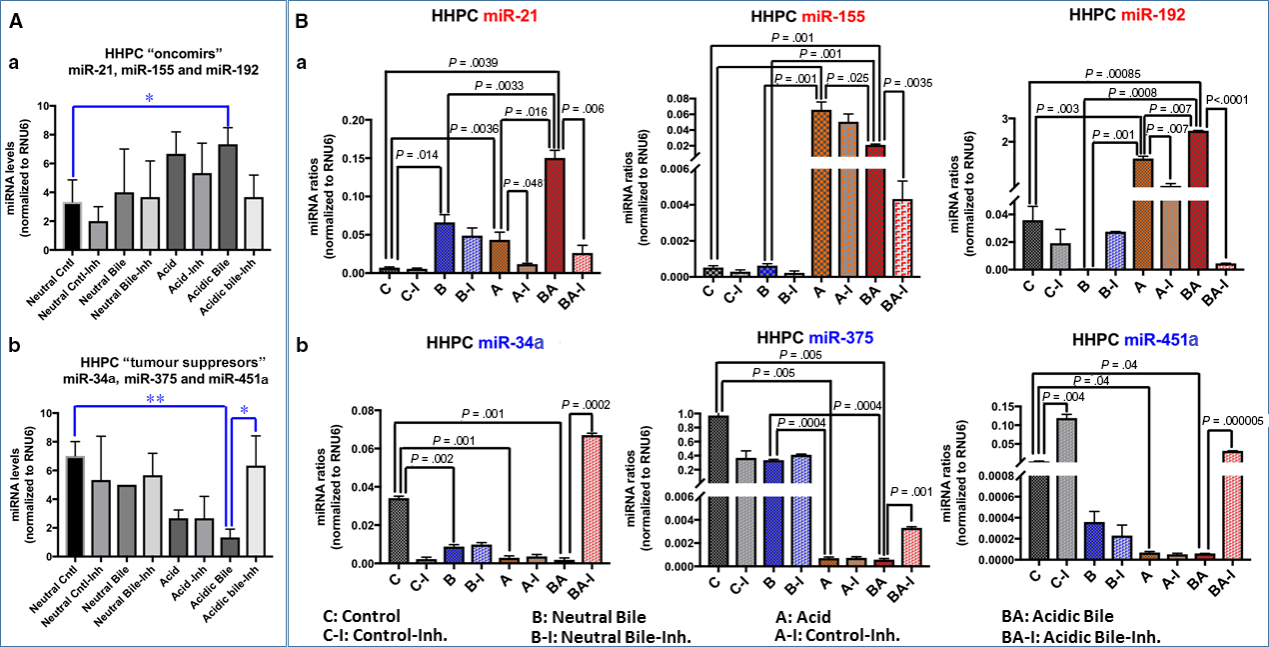
NF‐κB inhibitor (20 μmol/L ΒΑΥ 11‐7082) reverses the acidic bile‐induced deregulation of cancer‐related miRNA markers, in normal human hypopharyngeal primary cells (HHPC). A, Acidic bile induces in HHPC (a) an up‐regulation of the analysed “oncomirs” demonstrated by significantly higher miRNA levels, compared to controls, inverted by NF‐κB inhibitor (BAY 11‐7082). (b) Acidic bile induces a down‐regulation of the analysed “tumour suppressor” miRNAs, demonstrated by significantly lower expression levels, compared to controls, that is also inverted by NF‐κB inhibitor (BAY 11‐7082) in HHPC (one‐way ANOVA; by Friedman; *P < .05; **<*P* < .005; GraphPad Prism 6.0) B, Graphs depict the significantly (a) decreased expression levels of miR‐21, miR‐155 and miR‐192 and (b) increased expression levels of miR‐34a, miR‐375 and miR‐451a, in HHPC exposed to acidic bile with NF‐κB inhibitor (BAY 11‐7082), compared to HHPC exposed to acidic bile without NF‐κB inhibitor (*P* values by t test; mean ± SD; multiple comparisons by Holm‐Sidak; GraphPad Prism 6.0). (Normalization control: small RNA RNU6B). (Data were obtained from three independent experiments)

**Figure 3:**
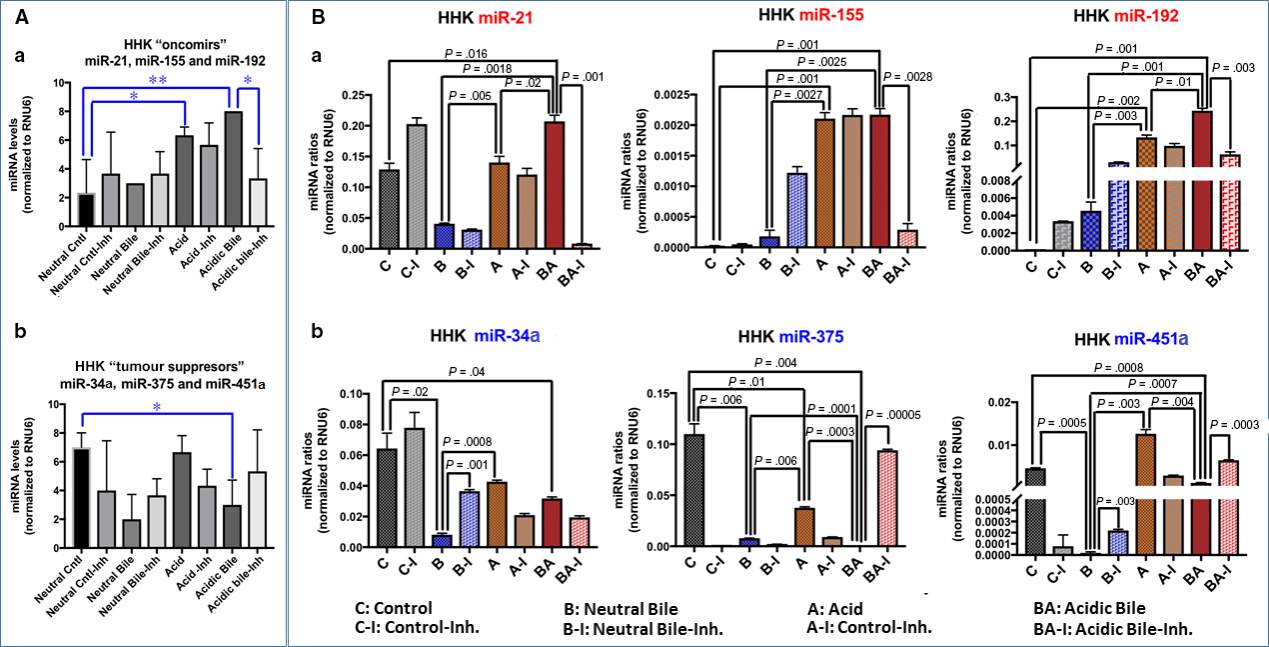
NF‐κB inhibitor (20 μmol/L ΒΑΥ 11‐7082) reverses the acidic bile‐induced deregulation of cancer‐related miRNA markers, in human hypopharyngeal keratinocytes (HHK). A, Acidic bile induces in HHK (a) an up‐regulation of the analysed “oncomirs”, demonstrated by significantly higher miRNA levels, compared to controls, inverted by NF‐κB inhibitor (BAY 11‐7082). (b) Acidic bile induces a down‐regulation of the analysed “tumour suppressor” miRNAs, demonstrated by significantly lower expression levels, compared to controls, that is also inverted by NF‐κB inhibitor (BAY 11‐7082) in HHK (one‐way ANOVA; by Friedman; *P < .05; **<*P* < .005; GraphPad Prism 6.0). B, Graphs depict the significantly (a) decreased expression levels of miR‐21, miR‐155 and miR‐192 and (b) increased expression levels of miR‐34a, miR‐375 and miR‐451a, in HHK exposed to acidic bile with NF‐κB inhibitor (BAY 11‐7082), compared to HHK exposed to acidic bile without NF‐κB inhibitor (*P* values by t test; mean ± SD; multiple comparisons by Holm‐Sidak; GraphPad Prism 6.0). (Normalization control: small RNA RNU6B). (Data were obtained from three independent experiments)

The in vitro effect of acid and bile combination in normal human hypopharyngeal cells selectively induced deregulation of cancer‐related miR‐21, ‐155, ‐192, ‐34α, ‐375 and ‐451α. NF‐κB inhibitor reversed the acidic bile (pH 4.0) induced deregulation of the analysed miRNA markers by preventing up‐regulation of “oncomirs” and inhibiting the down‐regulation of “tumour suppressor” miRNAs, in treated HHPC (Figure 2) and HHK (Figure 3).

#### 3.2.1 The effect of NF‐κB inhibitor reverses the acidic bile‐induced deregulation of cancer‐related miRNAs in treated normal human hypopharyngeal cells

Normal human hypopharyngeal cells, HHPC and HHK, treated with acidic bile, demonstrated a significant overexpression (up‐regulation) of the analysed “oncomirs” (*P *=* *.0047 and *P *=* *.0455, respectively) (Figures 2A‐a and 3A‐a) and a significant decrease (down‐regulation) of the analysed “tumour suppressor” miRNA levels (*P *=* *.0133 and *P *=* *.0046, respectively) (Figures 2A‐b and 3A‐b), compared to controls (one‐way ANOVA; by Friedman). In contrast, HHPC and HHK exposed to acidic bile with NF‐κB inhibitor demonstrated lower levels of “oncomirs” and significantly higher levels of “tumour suppressor” miRNAs, compared to those cells exposed to acidic bile without NF‐κB inhibitor (*P *<* *.05) (Figures 2 and 3) (one‐way ANOVA; by Friedman).

Human hypopharyngeal primary cells exposed to acidic bile with BAY 11‐7082 demonstrated a significant decrease in “oncomirs” miR‐21 (*P *=* *.006), miR‐155 (*P *=* *.0035) and particularly of miR‐192 levels (*P *<* *.00001) (Figure 2B‐a), as well as a significant increase in “tumour suppressor” miR‐34a (*P *=* *.00023), miR‐375 (*P *=* *.00134) and particularly of miR‐451a levels (*P *<* *.000001) (Figure 2B‐b), compared to HHPC exposed to acidic bile without NF‐κB inhibitor (*t* test analysis; multiple comparisons by Holm‐Sidak). We also observed that HHPC exposed to acid with BAY 11‐7082 demonstrated a significant decrease in miR‐21 and miR‐192 levels, compared to those exposed to acid without BAY 11‐7082 (*P *=* *.038 and *P *=* *.007, respectively) (Figure 2B‐a) (*t* test analysis; multiple comparisons by Holm‐Sidak).

Similarly, HHK exposed to acidic bile with BAY 11‐7082 exhibited a significant decrease in “oncomir” miR‐21 levels (*P *=* *.001265), miR‐155 (*P* = .002) and miR‐192 (0.003) (Figure 3B‐a), as well as a significant increase in “tumour suppressor” miR‐451a levels (*P *=* *.000339) and miR‐375 (*P* < .0001) (Figure 3B‐b), compared to HHK exposed to acidic bile without NF‐κB inhibitor (*t* test analysis; multiple comparisons by Holm‐Sidak). We also observed that HHK exposed to neutral bile with BAY 11‐7082 demonstrated a significant increase in miR‐34a (*P *=* *.0012) and miR‐451a (*P *=* *.0029) levels, relative to HHK expose to neutral bile without NF‐κB inhibitor (Figure 3B‐b). Finally, we observed that HHK exposed to acid with BAY 11‐7082 showed a trending reduction, without statistical significance, in oncomirs miR‐21 and miR‐192, compared to HHK exposed to acid without BAY 11‐7082 (Figure 3B‐a).

#### 3.2.2 NF‐κB inhibitor induces a reversed cancer‐related miRNA phenotype in acidic bile‐treated normal human hypopharyngeal cells, relative to controls

**Figure 4:**
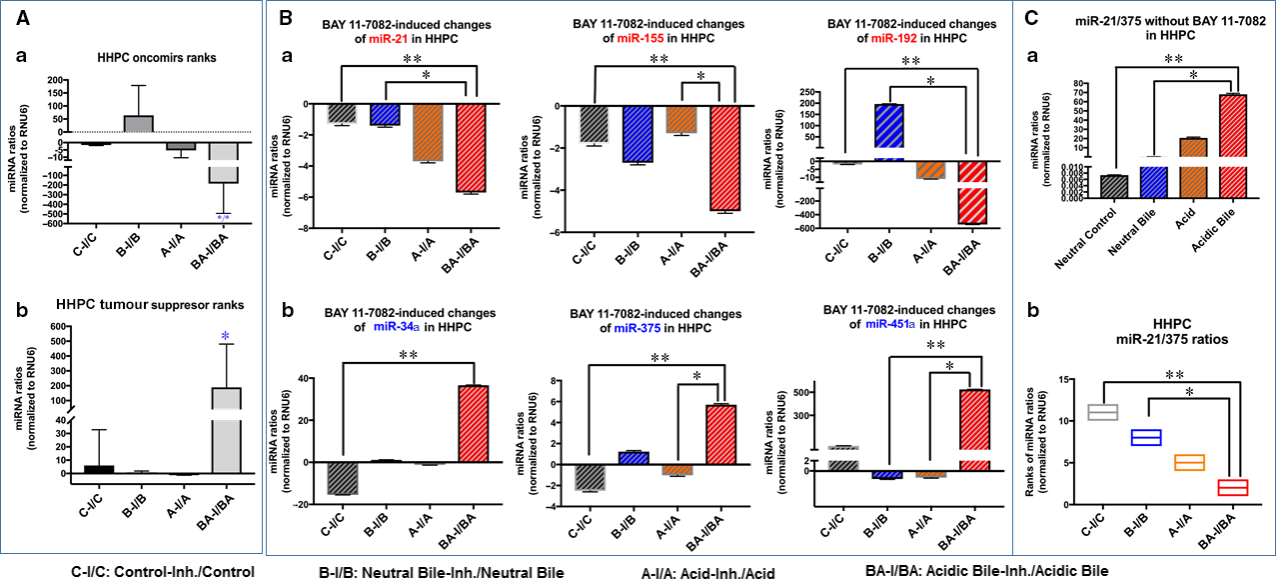
BAY 11‐7082‐induced miRNA phenotypes in acidic bile‐treated human hypopharyngeal primary cells (HHPC). A, NF‐κB inhibitor (BAY 11‐7082) in acidic bile‐treated HHPC induces a reversed phenotype of the analysed (a) “oncomirs” and (b) “tumour suppressor”, compared to neutral control and neutral bile (one‐way ANOVA; by Kruskal‐Wallis; *P < .05; GaphPad Prism 6.0). B, BAY 11‐7082 induces a reversed miRNA phenotype (with/without BAY 11‐7082) of each miR‐21, miR‐155, miR‐192, miR‐34*a*, miR‐375 and miR‐451*a*, in acidic bile exposure compared to other experimental‐ or control‐treated HHPC (one‐way ANOVA, by Kruskal‐Wallis; *P < .05; **P < .005; GraphPad Prism 6.0). C, (a) Acidic bile induces significantly higher miR‐21/375 ratios in HHPC, compared to neutral control or neutral bile. (b) BAY 11‐7082 induces significantly lower miR‐21/375 ratios (with/without BAY 11‐7082) in acidic bile‐treated HHPC, compared to neutral control or neutral bile (one‐way ANOVA, by Kruskal‐Wallis; *P < .05; **P < .005; GraphPad Prism 6.0). (Data were obtained from three independent experiments)

**Figure 5:**
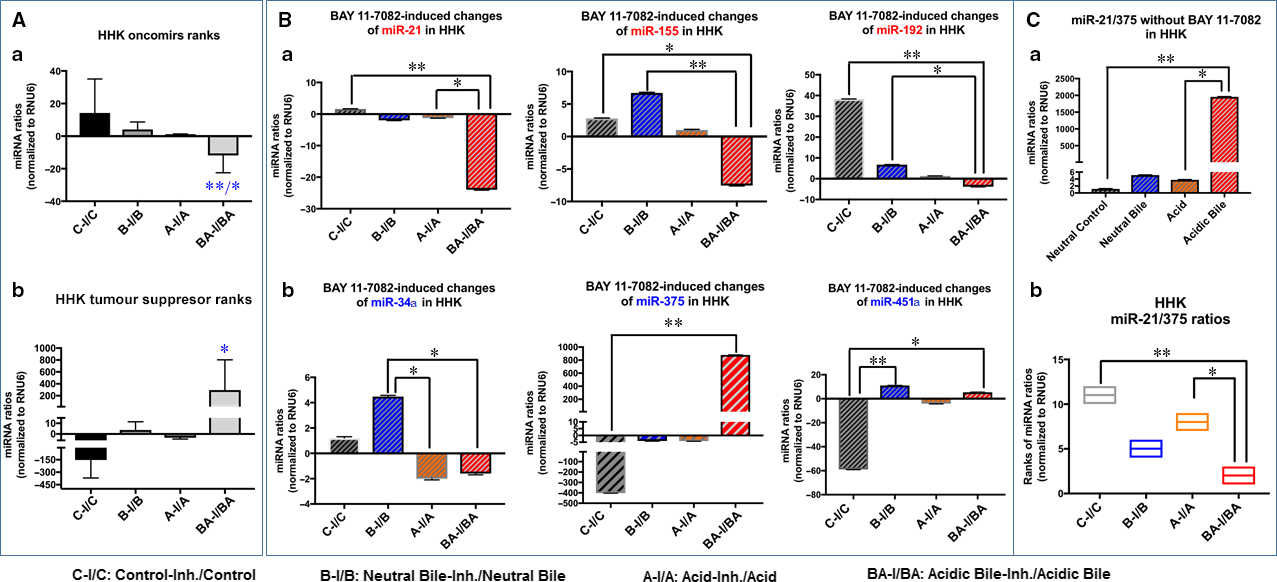
BAY 11‐7082‐induced miRNA phenotypes in acidic bile‐treated human hypopharyngeal keratinocytes (HHK). A, NF‐κB inhibitor (BAY 11‐7082) in acidic bile‐treated HHK induces a reversed phenotype (with/without BAY 11‐7082) of the analysed (a) “oncomirs” and (b) “tumour suppressor” miRNAs, compared to neutral controls or neutral bile. (one‐way ANOVA, by Kruskal‐Wallis; *P < .05; **P < .005; GraphPad Prism 6.0). B, BAY 11‐7082 induces a reversed miRNA phenotype (with/without BAY 11‐7082) of each miR‐21, miR‐155, miR‐192, miR‐34*a*, miR‐375 and miR‐451*a*, in acidic bile exposure compared to other experimental or control‐treated HHK. (one‐way ANOVA, by Kruskal‐Wallis; *P < .05; **P < .005; GraphPad Prism 6.0). C, (a) Acidic bile induces significantly higher miR‐21/375 ratios in HHK, compared to controls. (b) BAY 11‐7082 induces significantly lower miR‐21/375 ratios (with/without BAY 11‐7082) in acidic bile‐treated HHK, compared to controls (one‐way ANOVA, by Kruskal‐Wallis; *P < .05; **P < .005; GraphPad Prism 6.0). (Data were obtained from three independent experiments)

We observed that NF‐κB inhibitor induced an inverted miRNA phenotype of “oncomirs” and particularly of “tumour suppressor” miRNAs in acidic bile‐treated normal human hypopharyngeal cells, HHPC (Figure 4) and HHK (Figure 5), compared to control, demonstrating significant changes of the expression ratios (with/without BAY 11‐7082) between acidic bile and control groups (*P *=* *.0442 and *P *=* *.0139, respectively, by Friedman). The reversal of miRNA phenotypes, by NF‐κB inhibitor, was particularly intense in acidic bile‐treated HHPC, demonstrating a significant decrease in expression ratios of “oncomirs” (Figure 4A‐a) and increase in expression ratios of “tumour suppressor” miRNAs (with/without BAY 11‐7082), compared to control (*P *=* *.0269, one‐way ANOVA; by Kruskal‐Wallis) (Figure 4A‐b). We also observed an inverted miRNA phenotype, by NF‐κB inhibitor, in acidic bile‐treated HHK, demonstrating a significant decrease in expression ratios of “oncomirs” (*P *=* *.0114, one‐way ANOVA; by Kruskal‐Wallis) (Figure 5A‐a) and an increase in expression ratios of “tumour suppressor” miRNAs (with/without BAY 11‐7082), compared to control (Figure 5A‐b).

Specifically, we observed that each particular miRNA, including miR‐21, miR‐155, miR‐192, miR‐34a, miR‐375 and miR‐451a, was affected by NF‐κB inhibitor in HHPC (*P *<* *.005, by Kruskal‐Wallis) (Figure 4B). On the other hand, miR‐21 and miR‐375 were the most affected by the application of NF‐κB inhibitor in HHK, among the analysed miRNA markers, representing significant expression changes in acidic bile‐treated normal hypopharyngeal cells, compared to control (*P *=* *.0022, by Kruskal‐Wallis) (Figure 5B).

We further showed HHPC treated with acidic bile without BAY 11‐7082 demonstrated a significantly higher miR‐21/375 ratio, compared to neutral control or neutral bile, (*P *=* *.0022, and *P *=* *.0415, respectively, by Kruskal‐Wallis) (Figure 4B), and similarly HHK treated with acidic bile without NF‐κB inhibitor showed a significantly higher miR‐21/375 ratio compared to neutral control or acid alone (*P *=* *.0022 and *P *=* *.0415, respectively, by Kruskal‐Wallis). In contrast, we observed that BAY 11‐7082 induced a significant reduction in miR‐21/375 ratios in acidic bile‐treated HHPC, compared to neutral control and neutral bile (*P *=* *.0022 and *P *=* *.0415, respectively) (Figure 4C‐b), as well as in HHK, compared to neutral control and acid alone (*P *=* *.0022 and *P *=* *.0415, respectively) (Figure 5C‐b). These observations suggest that NF‐κB inhibition is capable of decreasing the acidic bile‐induced elevated miR‐21/375 ratios in both HHPC and HHK.

### 3.3 BAY 11‐7082 preferentially affects the acidic bile rather than the neutral bile‐induced cancer‐related miRNA phenotypes

We observed that miRNA changes induced by NF‐κB inhibitor included a significant difference between acidic bile‐ (pH 4.0) and neutral bile (pH 7.0)‐treated cells (Figure S1).

We observed that BAY 11‐7082 induced significantly lower expression ratios (with/without BAY 11‐7082) of “oncomirs”, miR‐21, miR‐155 and miR‐192, in acidic bile compared to neutral bile‐treated HHPC (*P *<* *.005) and HHK (*P *<* *.05) (Figure S1 A‐a, B). On the other hand, we found that BAY 11‐7082 induced significantly higher expression ratios (with/without BAY 11‐7082) of “tumour suppressor” miR‐34a, miR‐375 and miR‐451a, in acidic bile, compared to neutral bile‐treated HHPC (*P *<* *.0005) and higher ratios of “tumour suppressor” miR‐375 and miR‐451a, in acidic bile, compared to neutral bile‐treated HHK (*P *<* *.0005) (Figure S1 A‐b, B).

### 3.4 Correlations among BAY 11‐7082‐induced oncogenic miRNA expressions in treated normal human hypopharyngeal cells

We identified a significant positive correlation between BAY 11‐7082‐induced expression changes of miR‐21 and miR‐155 (*r *=* *.92390451, *P *=* *.0249), miR‐21 and miR‐192 (*r *=* *.9964257, *P *=* *.0003) and miR‐155 and miR‐192 (*r *=* *.95292381, *P *=* *.0121), in treated HHPC.

We also identified a significant positive correlation between BAY 11‐7082‐induced expression changes of “tumour suppressor” miR‐34a and miR‐375 (*r *=* *.99320292, *P *=* *.0007), miR‐34a and miR‐451a (*r *=* *.92967608, *P *=* *.0221) and between miR‐375 and miR‐451a (*r *=* *.88047938, *P *=* *.0487 in treated HHPC.

We found a strong inverse correlation between BAY 11‐7082 induced expression changes of miR‐21 and miR‐375 (*r *=* *−.990359, *P *=* *.0096) of different treated groups of normal human hypopharyngeal cells (both HHK and HHPC) (Figure S2). We observed a strongly inverted correlation between BAY 11‐7082‐induced expression changes (with/without BAY 11‐7082) of “oncomirs” miR‐155 or miR‐192 and “tumour suppressor” miR‐451a in HHPC (*r *=* −*.914115213, *P *=* *.0298 and *r *=* *−.995811529, *P *=* *.0003, respectively), as well as miR‐155 and miR‐375 in HHK (*r *=* *−.989308022, *P *=* *.0107), as well as of miR‐192 and miR‐451a in HHK (*r *=* *−.996114636, *P *=* *.0039) (Figure S2).

### 3.5 Correlations between BAY 11‐7082‐induced changes of oncogenic miRNA markers and NF‐κB‐related genes in treated human hypopharyngeal primary cells

To determine the correlations between BAY 11‐7082‐induced expression changes (with/without BAY 11‐7082) of the analysed oncogenic miRNA markers and NF‐κB‐related genes, in the same treated groups, we performed qPCR analysis from the same pool of total RNA.

Pearson analysis revealed significant relationships between BAY 11‐7082‐induced changes (with/without NF‐κB inhibitor) in miRNA levels of the analysed markers and mRNA levels of NF‐κB‐related genes [RELA(p65), TNF‐α, IL‐1β, IL‐6 and STAT3] (Table S3) and in treated HHPC. Specifically, a strongly positive correlation was found between BAY 11‐7082‐induced expression changes of (i) miR‐192 and RELA(p65) (*r *=* *.99944615, *P *=* *.0005), STAT3 (*r *=* *.9957416, *P *=* *.0003), TNF‐α (*r *=* *.9893677, *P *=* *.0013) and IL‐1β (*r *=* *.9598826, *P *=* *.0096), as well as (ii) miR‐155 and RELA(p65) (*r *=* *.9266003, *P *=* *.0236), STAT3 (*r *=* *.9216948, *P *=* *.0260), TNF‐α (*r *=* *.9039464, *P *=* *.0352) and NF‐κB‐related cytokines, IL‐6 (*r *=* *.99554516, *P *=* *.0045) and IL‐1β (*r *=* *.97907598, *P *=* *.0209), in treated HHPC.

Alternatively, a strongly inverted correlation was observed between BAY 11‐7082‐induced expression changes of (i) miR‐34a and RELA(p65) (*r *=* *−.9462625, *P *=* *.0148), STAT3 (*r *=* *−.9444849, *P = *.0156), TNF‐α (*r *=* *−.8868372, *P *=* *.0449), IL‐6 (*r *=* *−.9299620, *P *=* *.0220) and IL‐1β (*r *=* *−.88470515, *P = *.0462); (ii) miR‐375 and RELA(p65) (*r *=* *−.9232761, *P *=* *.0252), STAT3 (*r *=* *−.9192839, *P *=* *.0272), IL‐6 (*r *=* *−.9789924, *P = *.0036) and IL‐1β (*r *=* *−.892283, *P = *.0417); as well as between (iii) miR‐451a and RELA(p65) (*r *=* *−.9972300, *P = *.0002), STAT3, (*r *=* *−.99762108, *P = *.0001), TNF‐α (*r *=* *−.99693963, *P* = .0002) or IL‐1β (*r *=* *−.98148507, *P *=* *.0030), in treated HHPC.

### 3.6 BAY 11‐7082 inhibits acidic bile‐induced nuclear translocation of p‐NF‐κB and oncogenic p‐STAT3 in human hypopharyngeal primary cells

**Figure 6:**
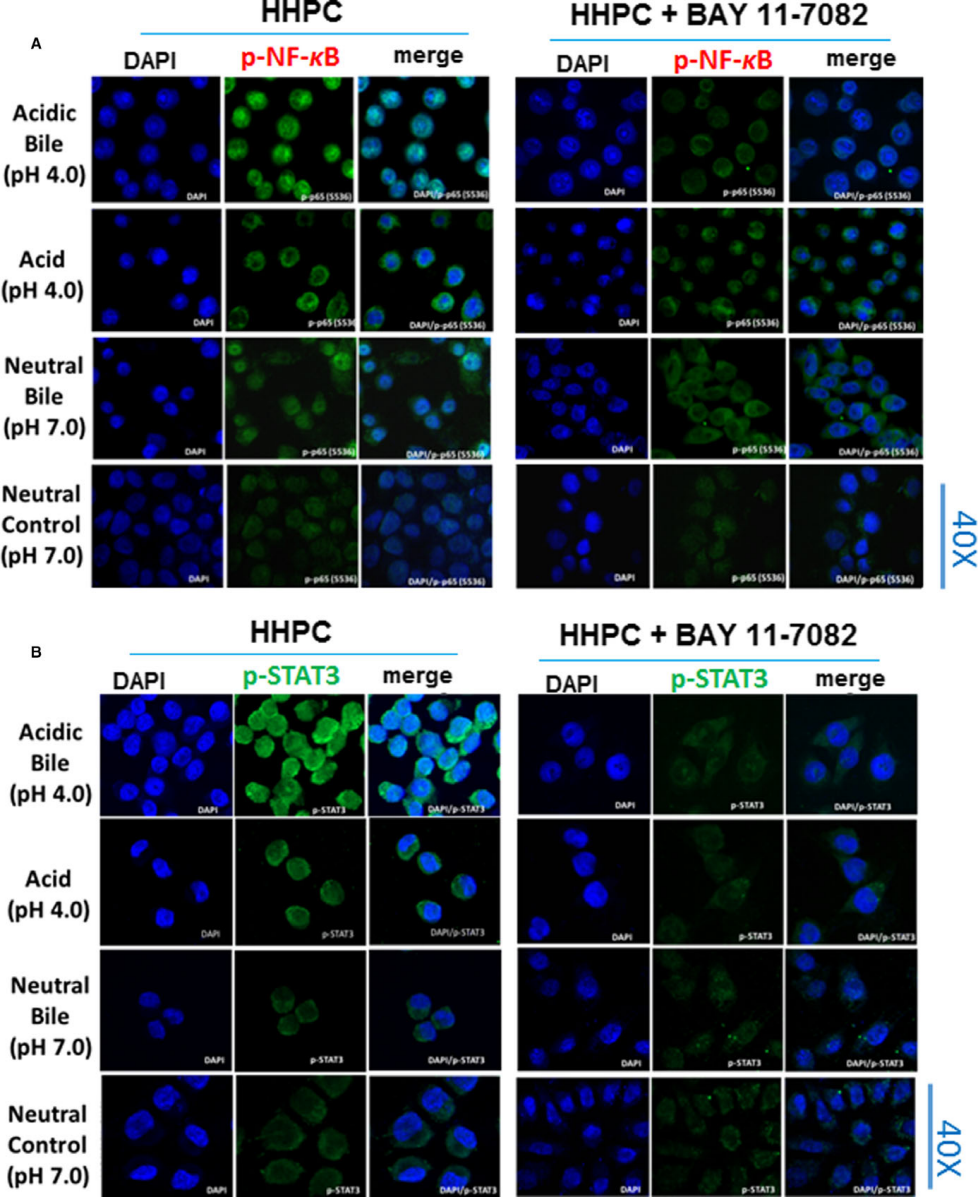
BAY 11‐7082 inhibits bile‐induced nuclear translocation of phospho‐NF‐κB and phospho‐STAT3 in normal human hypopharyngeal cells. Immunofluorescence staining of (A) phospho‐NF‐κB (p‐p65 S536) and (B), phospho‐STAT3 (Tyr705) reveals that application of BAY 11‐7082 particularly inhibits the p‐NF‐κB and p‐STAT3 nuclear translocation in acidic bile‐treated human hypopharyngeal primary cells (HHPC), demonstrating decreased p‐p65 and p‐STAT3 nuclear staining [green: p‐p65 (S536) or pSTAT3 (Tyr705); blue: DAPI for nuclear staining]

STAT3, a crucial gene in progression of HNSCC, has been previously suggested to up‐regulate miR‐21, in a NF‐κB‐dependent form of IL‐6 up‐regulation.^34^ Our data from immunofluorescence assay demonstrated that the acidic bile‐induced activated STAT3 and NF‐κB were inhibited by NF‐κB inhibitor in treated HHPC (Figure 6). Specifically, we observed that acidic bile‐treated HHPC showed an intense nuclear staining for both phospho‐NF‐κB (p‐p65 S556) (Figure 6A) and phospho‐STAT3 (Tyr705) (Figure 6B) that was inhibited by BAY 11‐7082. Neutral bile and acid alone induced a less intense nuclear staining of p‐p65 and p‐STAT3, compared to acidic bile‐treated cells, that was also inhibited by BAY 11‐7082. Neutral control‐treated HHPC showed a weak nuclear staining of both p‐STAT3 and p‐p65, while BAY 11‐7082 induced a minimal change. Our findings suggest that oncogenic STAT3 was particularly activated in acidic bile‐treated cells, as previously demonstrated in 45‐day acidic bile‐treated murine laryngopharyngeal mucosa with pre‐malignant lesions and deregulations of cancer‐related miRNA markers. STAT3 activation provides further evidence for an acidic bile effect mediated by interactions of activated NF‐κB with oncogenic miRNA.

### 3.7 NF‐κB mediates acidic bile‐induced interactions between cancer‐related miRNA markers and NF‐κB‐related genes with oncogenic function in acidic bile‐treated human hypopharyngeal primary cells

**Figure 7:**
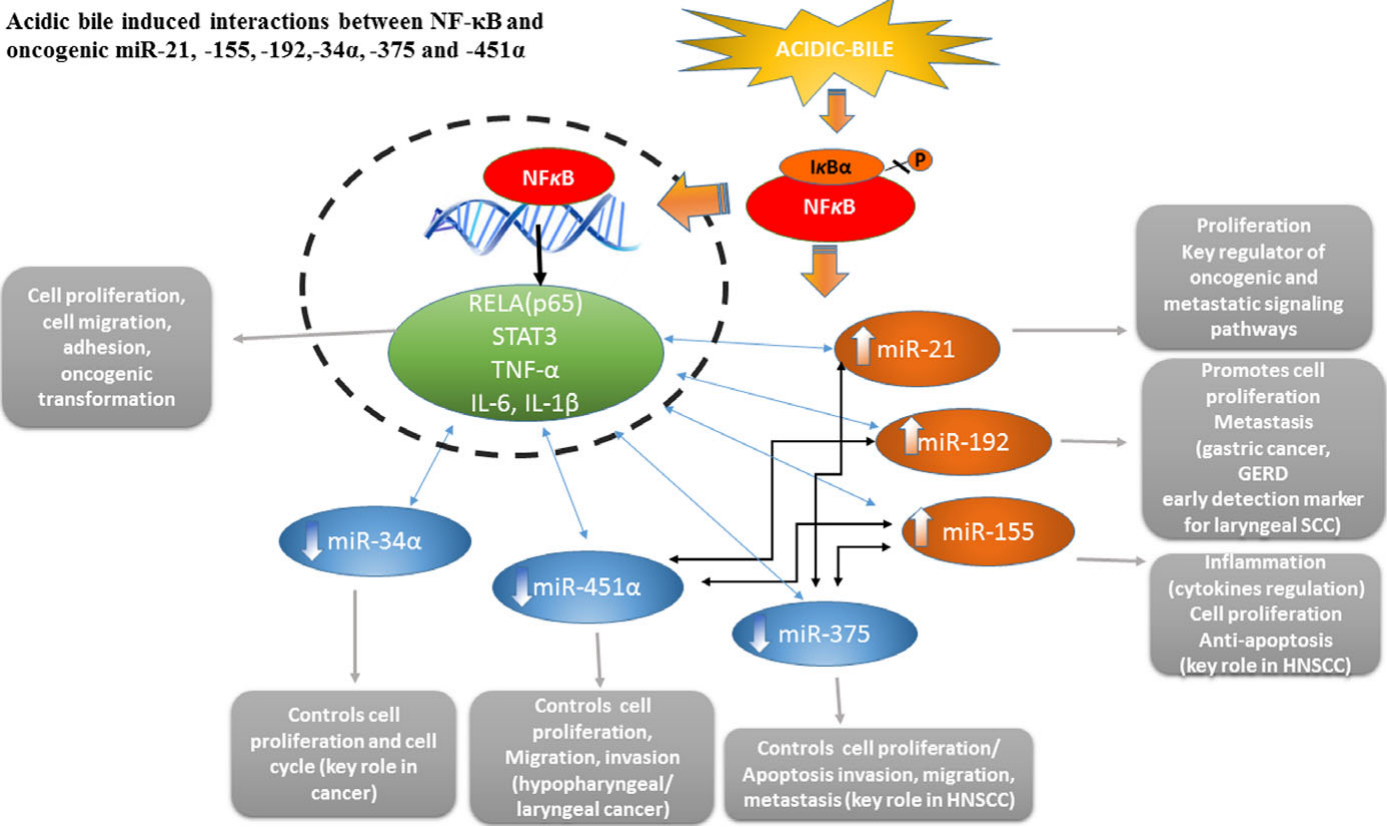
Acidic bile‐induced interactions between NF‐κB and oncogenic miR‐21, ‐155, ‐192, ‐34α, ‐375 and ‐451α markers, in treated human hypopharyngeal primary cells and their role in hypopharyngeal carcinogenesis

Figure 7 summarizes the observed acidic bile‐induced deregulation of oncogenic miRNA markers (as demonstrated in Figures 2, 3, 4, 5) that is prevented by NF‐κB inhibitor (BAY 11‐7082). Additionally, Figure 7 demonstrates proposed interactions among cancer‐related miRNA markers and NF‐κB‐related genes (Table S3), supported by the observed significant correlations among their expression changes.

### 3.8 BAY 11‐7082 reduces cell viability of the acidic bile‐treated normal human hypopharyngeal cells

We performed a cell viability assay, in bile‐treated normal human hypopharyngeal cells (HHPC and HHK) at pH 4.0 and pH 7.0, compared to controls (pH 4.0 and pH 7.0). NF‐κB inhibitor, BAY 11‐7082, exhibited strong negative effects on cell viability of the acidic bile (pH 4.0)‐treated HHPC and HHK, as shown by the significantly reduced percentages of viable cells after exposure to BAY 11‐7082 (*P* < .05, multiple *t* test) (Figure S3). On the other hand, NF‐κB inhibitor exhibited a weak negative effect on cell viability of bile‐treated HHPC and HHK at pH 7. DMSO had no negative effects on cell viability of treated cells, indicated by similar percentages of viable cells compared to controls (Figure S3).

## 4 DISCUSSION

Nuclear factor kappa B (NF‐κB) is a key factor that mediates inflammatory and early tumorigenic events in epithelial cells,^35^ and its importance in initiation and progression of cancer, including head and neck cancer, has been widely supported 23, 36, 37, 38, 39, 40, 41 by its interactions with a complex network of other cancer‐related transcriptional factors, cytokines and growth factors.^34^, ^42^, ^43^, ^44^, ^45^, ^46^ Additionally, Van Waes and Chen recently showed a cluster of genes and miRNA markers that are related to activated NF‐κB and that may contribute to an aggressive phenotype of head and neck cancer.^23^, ^38^

Here, we present the first in vitro report that bile and acid combination deregulates cancer‐related miRNA markers in normal human hypopharyngeal cells and that a pharmacologic inhibitor, BAY 11‐7082, is capable of reversing the acidic bile‐induced miRNA phenotypes. Our current findings demonstrate that, among the analysed miRNA markers, miR‐21 and miR‐375 are the most affected by the NF‐κB inhibitor, underscoring the role of activated NF‐κB with miR‐21 and miR‐375, in promoting acidic bile‐induced cancer‐related molecular alterations in hypopharyngeal cells. There is further evidence that microRNA markers, such as “oncomir” miR‐21 and “tumour suppressor” miR‐375, play a crucial role in initiation and progression of HNSCC.^10^, ^12^, ^13^ Arantes LMRB et al recently reported the fundamental role of miR‐21, as a biomarker, in head and neck carcinogenesis,^47^ while miR‐375 has been proposed as a predictive biomarker for early diagnosis in laryngeal cancer.^48^

Yang et al demonstrate that NF‐κB up‐regulates the expression of miR‐21, by binding to its gene promoter.^49^ Other studies also document NF‐κB binding sites on the promoter of miR‐21.^34^, ^50^ Furthermore, STAT3, a crucial gene in progression of HNSCC,^40^, ^41^ has also been previously implicated in up‐regulation of miR‐21, in a manner of NF‐κB‐dependent IL‐6 up‐regulation.^34^ The NF‐κB/IL‐6/STAT3/miR‐21 interaction therefore appears to be supported by the effect of BAY 11‐7082 in down‐regulating the expression of these oncogenic factors.

Whereas the ratio of miR‐21/375 has been considered a potential biomarker related to poor prognosis of supraglottic cancer,^13^, ^14^ our data demonstrate a significant increase in miR‐21/375 ratios in cells exposed to acidic bile (pH 4.0), compared to controls, a relationship that is effectively inverted in the presence of NF‐κB inhibitor, again strongly supporting the miR‐21/375 ratio, as a potential biomarker in acidic bile‐induced cancer‐related molecular events in hypopharyngeal cells, mediated by NF‐κB pathway.

Our novel findings also demonstrate that NF‐κB inhibition significantly prevents the acidic bile‐induced up‐regulation of miR‐155. Gerloff et al showed that NF‐κB can up‐regulate miR‐155 by binding on its promoter.^51^ Others have shown that constitutive up‐regulation of miR‐155 may mediate prolonged inflammatory reactions leading to cancer.^18^, ^52^ We previously showed a significant up‐regulation of miR‐155 in laryngopharyngeal mucosa treated by acidic bile accompanied by pre‐neoplastic lesions.^10^ Our current findings show that BAY 11‐7082‐induced miR‐155 levels resulted in strong positive correlations with pro‐inflammatory TNF‐α, IL‐1β and ΙL‐6 mRNAs, suggesting a protective role of NF‐κB inhibition in pro‐inflammatory events linked to downstream oncogenic pathways.

Our novel findings also showed that BAY 11‐7082 suppressed the acidic bile‐induced miR‐192 levels in treated human hypopharyngeal cells with strong positive correlations between BAY 11‐7082‐induced miR‐192 levels and inflammatory and key cancer molecules, such as RELA(p65), STAT3 and TNF‐α. Up‐regulation of “oncomir” miR‐192 has been previously linked to GERD 53, 54 and associated with supraglottic laryngeal cancer and metastasis.^15^

Our current findings also demonstrate that miR‐451a could be an important marker of acidic bile‐related laryngopharyngeal carcinogenesis, in agreement with Fukumoto et al who previously suggested miR‐451a is a tumour suppressor marker in hypopharyngeal SCC.^55^ This in vitro measure is in line with our 45‐day in vivo model where a significant down‐regulation of miR‐451a occurred.^10^

We also showed a significant effect of NF‐κB inhibition on “tumour suppressor” miR‐34a, in human hypopharyngeal primary cells, under the exposure of acidic bile. MiR‐34a is a known tumour suppressor and key regulator miRNA in HNSCC,^17^, ^23^ and our prior in vivo findings showed that acidic bile down‐regulated miR‐34a levels in treated laryngopharyngeal mucosa. Although previous studies reported NF‐κB binding sites on the promoter of miR‐34a,^21^, ^56^ and an increase in miR‐34a levels in oesophageal cells under NF‐κB activation,^21^ the exact mechanism of miR‐34a regulation by NF‐κB is not yet obvious. Alternatively, it has been shown that STAT3 can directly repress miR‐34a, while an active IL‐6R/STAT3/miR‐34a loop was found necessary for EMT, invasion and metastasis of colorectal cancer cell line.^56^ Our current data support the notion that NF‐κB inhibition is capable of preventing down‐regulation of miR‐34a by acidic bile.

As would be expected, the individual response of HHPC and HHK was not always congruent. The ability of BAY 11‐7082 to reverse acidic bile‐induced phenotype of “oncomir” miR‐192 was more prominent in HHPC compared to HHK. Likewise, the effect of NF‐κB inhibition regarding “tumour suppressor” miRNAs miR‐34a and miR‐451a was more intense in HHPC relative to HHK under acidic bile exposure. We are of the understanding that effects may be related to differences in maturational status. Primary cells HHPC, considered to be less mature and more sensitive to injurious external stimuli than immortalized keratinocytes HHK, may respond differently to NF‐κB inhibition. The miRNA phenotype observed in HHPC was in fact similar to that observed in vivo, an observation in support of this view.^10^

Our novel data demonstrate strong inverted correlations among the BAY 11‐7082‐induced levels of the analysed tumour suppressors” miR‐34a, miR‐375 and miR451a, and NF‐κB‐related genes, such as RELA(p65), STAT3, TNF‐α, IL‐6 and IL‐1β, that previous studies documented as crucial mediators of inflammatory and neoplastic events in head and neck cancer^35^, ^36^, ^37^, ^38^, ^39^, ^40^, ^41^, ^42^, ^43^, ^44^, ^45^, ^46^ (Figure 7). In line with our recent study,^33^ our current data suggest that NF‐κB inhibition may reverse acidic bile‐induced molecular events in normal human hypopharyngeal cells that are known to link inflammation to cancer, thereby in a sense shielding HHPC from the effects of bile‐induced oncogenic molecular events.

We showed that BAY 11‐7082 is also capable of preventing acid alone‐induced deregulations of miR‐21 and miR‐192, but not miR‐155 or “tumour suppressor” miRNAs in HHPC, suggesting that NF‐κB inhibition could reverse a part of acid‐induced miRNA phenotype in normal human hypopharyngeal cells. Although acid alone may up‐regulate selected “oncomirs”, it was not capable of accelerating activation of oncogenic STAT3 or other cancer‐related molecular events. Similarly, bile at neutral pH seemed to contribute to deregulations of “tumour suppressor” miRNAs, such as miR‐34a and miR‐451a, but was not capable of accelerating activation of oncogenic STAT3 or other cancer‐related molecules. Our data support the observation that acid and bile in combination but not acid or bile alone may contribute to cancer‐related molecular events, mediated by NF‐κB.

Our findings revealed that NF‐κB inhibition resulted in a significant reduction in viable cells, particularly in acidic bile‐treated groups. The identified decreased cell viability induced by BAY 11‐7082 is in line with previous studies.^27^ The preferential effect of BAY 11‐7082 in acidic bile‐treated groups is especially interesting because it suggests its effect on cell viability is not global but related to specific events, in those groups. Interestingly, other studies have shown that increased expression of miR‐34a and miR‐375 reduces cell viability of cancer cell lines.^57^, ^58^ In this regard, our present data also showed that BAY 11‐7082 decreases the levels of “oncomirs”, miR‐155, miR‐192 and miR‐21 levels, while increasing the levels of “tumour suppressors”, miR‐34a and miR‐375, raising the view that specific cancer‐related miRNAs interactions may be at least partially responsible for decreased cells viability.

## 5 CONCLUSION

Our novel findings from an in vitro model of normal human hypopharyngeal cells demonstrate that NF‐κB inhibitor significantly reverses the acidic bile‐induced deregulations of miRNA markers with oncogenic function. The application of the pharmacologic NF‐κB inhibitor BAY 11‐7072 on normal human hypopharyngeal cells effectively prevents the acidic bile‐induced up‐regulation of “oncomirs” miR‐21, miR‐155 and miR‐192 and down‐regulation of “tumour suppressors” miR‐34a, miR‐375 and miR‐451a, strongly supporting NF‐κB as an important key molecule in acidic bile‐induced molecular events in hypopharyngeal cells. We present strong interactions among the BAY 11‐7082‐induced expression levels of the analysed cancer‐related miRNA markers and well‐known mediators of inflammatory and oncogenic pathways, including RELA(p65), TNF‐α and IL‐6 or IL‐1β and oncogenic STAT3, suggesting a protective role of NF‐κB inhibition in acidic bile‐induced inflammatory events linked to cancer. The fact that NF‐κB inhibition strongly reverses the acidic bile‐induced miR‐21/375 ratio and miR‐192 and miR‐451a levels further supports their close relationship with the NF‐κB‐activated pathway, emphasizing their use as potential biomarkers of acidic bile‐related effect. Future investigation using pharmacologic or non‐pharmacologic NF‐κB inhibitors, such as dietary analogues in in vivo models may reveal the capability of NF‐κB inhibition in preventing or reversing the acidic bile‐induced deregulations of cancer‐related miR‐21, miR‐21, miR‐155, ‐192, 34a, ‐375 and ‐451a in treated hypopharyngeal mucosa.

## CONFLICT OF INTEREST

The authors whose names are listed in this article certify that they have no affiliations with or involvement in any organization or entity with any financial interest or non‐financial interest in the subject matter or materials discussed in this manuscript.

## AUTHOR CONTRIBUTIONS

DV, SD and CTS contributed to conceptualization and data curation. SD and DV performed formal analysis. CTS acquired funding. SD, DV and CTS contributed to investigation. DV contributed to methodology. CTS administered the project. CTS and DV collected resources. DV developed software. DV and CTS supervised the study. DV, SD and CTS validated the study. DV contributed the visualization. DV, SD and CTS wrote the original manuscript. CTS, DV and SD wrote, reviewed and edited the manuscript.
