# Supplementary figures and images for: NF‐κB inhibition reverses acidic bile‐induced miR‐21, miR‐155, miR‐192, miR‐34a, miR‐375 and miR‐451a deregulations in human hypopharyngeal cells
Source: J Cell Mol Med. 2018 Mar 8;22(5):2922–34. doi: 10.1111/jcmm.13591 (PMC5908126; doi:10.1111/jcmm.13591)

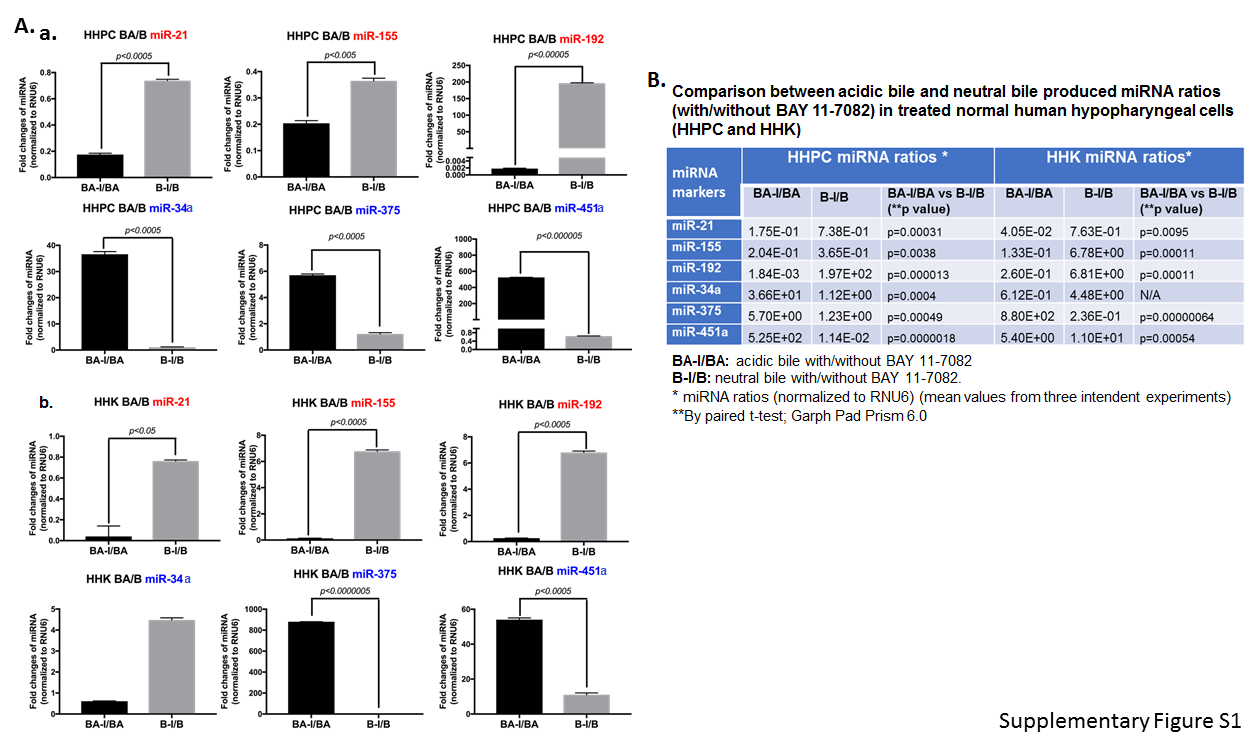

Supplement: Supplementary file 1 [file JCMM-22-2922-s001.tiff]

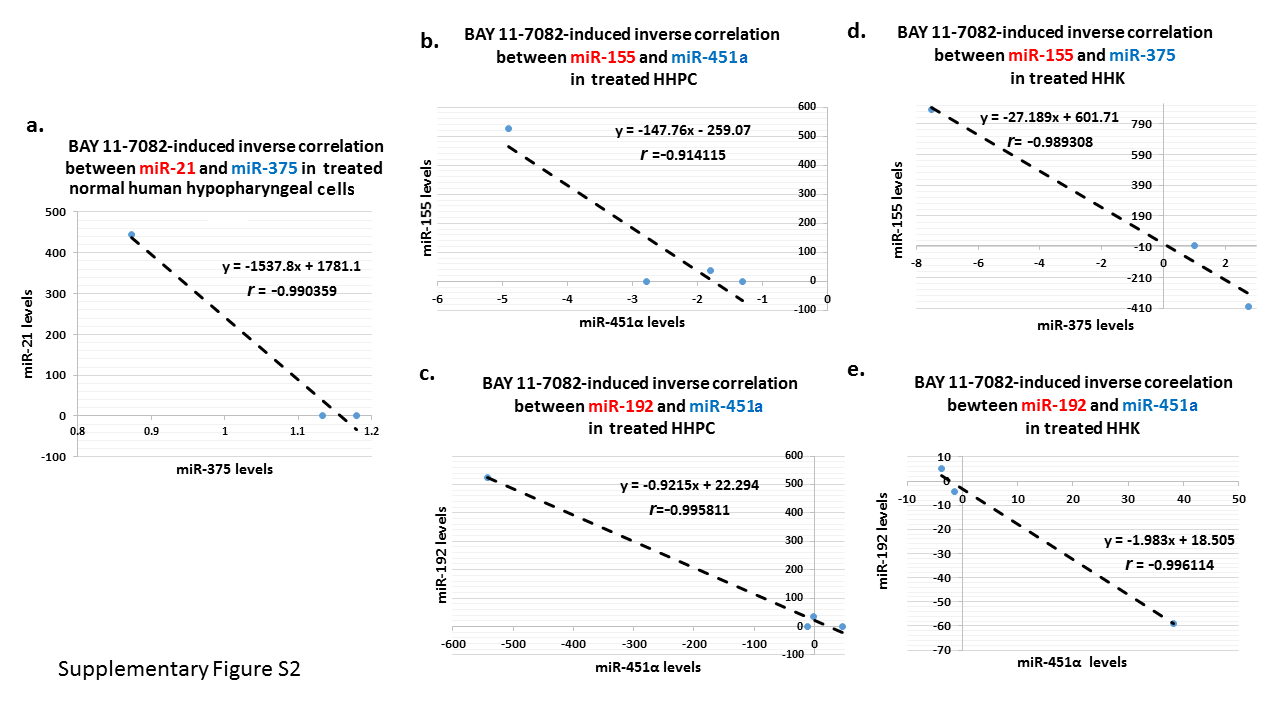

Supplement: Supplementary file 2 [file JCMM-22-2922-s002.tiff]

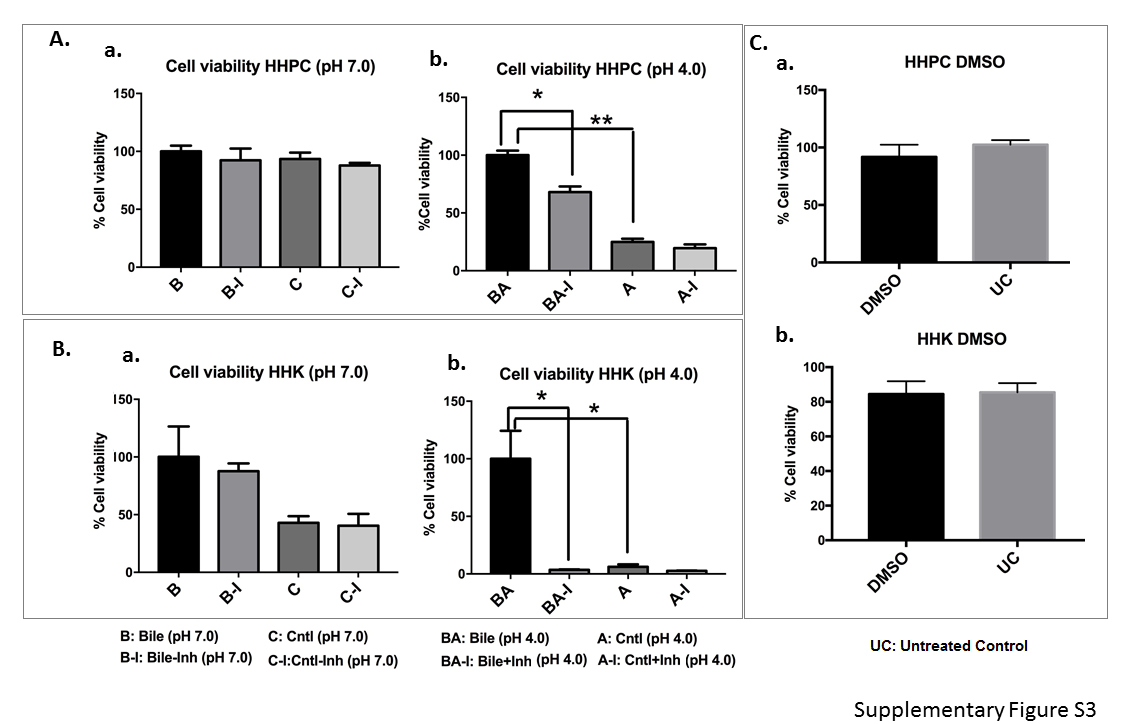

Supplement: Supplementary file 3 [file JCMM-22-2922-s003.tiff]
