# Supplementary material for: NF‐κB inhibition reverses acidic bile‐induced miR‐21, miR‐155, miR‐192, miR‐34a, miR‐375 and miR‐451a deregulations in human hypopharyngeal cells
Source: J Cell Mol Med. 2018 Mar 8;22(5):2922–34. doi: 10.1111/jcmm.13591 (PMC5908126; doi:10.1111/jcmm.13591)
Supplement: Supplementary file 4 [file JCMM-22-2922-s004.docx]

**Title: NF-*κ*B inhibition reverses acidic bile-induced miR-21, miR-155, miR-192, miR-34a, miR-375 and miR-451a deregulations in human hypopharyngeal cells**

Sotirios G. Doukas^1^, Dimitra P. Vageli^1^ and Clarence T. Sasaki^1^*

^1^The Yale Larynx laboratory, Department of Surgery, Yale School of Medicine, New Haven, CT, USA

## Supplementary Information

## Supplementary Methods

**Cell culture**

***Human hypopharyngeal primary cells:***

Human hypopharyngeal primary cells (HHPC) (2^nd^ passage) from Celprogen Inc. CA, USA were grown in Human Hypopharyngeal Normal Cell Culture Media with Serum (Celprogen Inc. CA, USA), at 37°C in humidified air and 5% CO2 and passed after reaching ~90% confluence, using 0.05% trypsin-EDTA (Gibco®, NY, USA). Media were gradually replaced by Serum Free Media (Celprogen Inc. CA, USA) and cells serum starved for 24 h before treatment.

***Human hypopharyngeal keratinocytes***

We used telomerase-immortalized human hypopharyngeal keratinocytes (HHK) (4^th^ passage), by expression of hTERT, extending their life span without altering the characteristic phenotypic properties of the cells, as previously described [8]. The cells were grown in keratinocyte serum free basal medium (KGM-2 SF, Gibco®, NY, USA) supplemented by L-Glutamine, BPE, hEGF and gentamicin (Gibco®, NY, USA), at 37°C in humidified air and 5% CO2.

***In vitro* exposure to acidic bile with and without BAY 11-7082**

***Acidic bile treatment***

We performed a repetitive exposure of HHPC (2^nd^ passage) and HHK (4^th^ passage) to bile (pH 4.0 and pH 7.0) and corresponding controls (pH 4.0 and pH 7.0), for 10-15 min, 3 times per day, for 5 days [8]. Bile fluid consisted of a mixture of conjugated bile salts of Glycocholic acid, Taurocholic acid, Glycochenodeoxycholic acid, Taurochenodeoxycholic acid, Glycodeoxycholic acid and Taurodeoxycholic acids (Sigma, St. Louis, MO and Calbiochem, San Diego, CA; USA) at molar concentration (20:3:15:3:6:1) as previously described [8], and considered to be close to “physiologic” [29,30].

We used two experimental and two corresponding control groups, as follows:

Experimental groups included HHPC and HHK repetitively exposed to bile at pH 4.0, the cut off of reflux disease [31,32], and at pH 7.0, as follows: (a) acidic bile (pH 4.0), containing 400 μM of bile, in full growth medium (Dulbecco modified Eagle’s medium/F12 10% FBS, 1% pen/strep, Gibco®, NY, USA), brought to pH 4.0 with 1M HCl (using a pH meter) and (b) neutral bile (pH 7.0), containing the same bile salts mixture in DMEM/F12 10% FBS, at pH 7.0.

Control groups included HHPC and HHK repetitively exposed to acid alone (pH 4.0) considered a positive control and neutral fluid (pH 7.0), considered a reference control, as follows: (a) acid control (pH 4.0), full growth DMEM/F12 10% FBS, brought to pH 4.0 with 1M HCl, and (b) neutral control (pH 7.0), full growth DMEM/F12 10% FBS, pH 7.0.

At the end of each treatment the media were removed and replaced with serum free media until the next exposure cycle [Human Hypopharyngeal Normal Cell Culture Media Serum Free, for HHPC cells; (Celprogen Inc. CA, USA), and KGM-2 SF, for HHK cells (Gibco®, NY, USA)].

***Acidic bile + BAY-11-7082***

In parallel with acidic bile treatment, we performed an additional procedure of repetitive exposure of HHPC (2^nd^ passage) and HHK (4^th^ passage) to acidic bile with BAY 11-7082, a pharmacologic inhibitor of NF-*κ*B (Calbiochem © 2016 EMD Millipore Corporation; Germany) [11], for 10-15 min, 3 times per day, for 5 days.

Experimental groups included an identical procedure of repetitive exposure of HHPC and HHK to bile at pH 4.0 and 7.0, as described above, in combination with BAY 11-7082 at final concentration of 20 μM of BAY 11-7082 (dilution from 100 mM stock solution in DMSO), as follows: (a) acidic bile plus Inhibitor (pH 4.0), containing 400 μM of acidic bile, in full growth medium (Dulbecco modified Eagle’s medium/F12 10% FBS, 1% pen/strep, Gibco®, NY, USA), and 20 μM of BAY 11-7082, brought to a pH of 4.0 with 1M HCl (using a pH meter) and (b) neutral bile plus Inhibitor (pH 7.0), containing the same bile salts mixture in DMEM/F12 10% FBS, at pH 7.0 and 20 μM of BAY 11-7082.

Control groups included a repetitive exposure to acid alone (pH 4.0) and neutral control (pH 7.0) in combination with BAY 11-7082, as follows: (a) acid control (pH 4.0), full growth medium, as described above, and 20 μM of BAY 11-7082, brought to pH 4.0 with 1M HCl and (b) neutral control (pH 7.0), full growth medium, as described above, and 20 μM of BAY 11-7082. We also used untreated cells as negative control and DMSO treated groups, as reference control for the NF-*κ*B inhibitor vehicle. DMSO was used at concentrations similar to those used for BAY 11-7082 solubilisation.

At the end of each treatment, the experimental and control media were removed and replaced with serum free media until the next exposure cycle, as described above.

At the end of treatment media were removed and cells or cell extracts were analysed.

***Immunofluorescence***

We performed an immunofluorescence (IF) assay, to explore the effect of NF-*κ*B inhibitor on the acidic bile-induced nuclear translocation of phospho-NF-*κ*B (p65, Ser536), and phospho-STAT3 (Tyr705). HHPC were grown on slides (multiwall chamber slides; Lab-Tek®) and underwent repeated exposure with experimental and control fluids with or without NF-*κ*B inhibitor (20 μM of BAY 11-7082). At the end of treatment, cells were fixed immediately after the final treatment in 4% paraformaldehyde (Sigma-Aldrich) for 7 minutes and incubated with 1:65 of primary anti-NF-*κ*B (rabbit polyclonal anti-phospho-p65 Ser536, AbD Serotec, BIO-RAD, CA, USA), or 1:100 of primary anti-phosho-STAT3 (Tyr705)(D3A7) XP Rabbit mAb; Cell signaling, Techn.) overnight at 4°C, after permeabilization of cell membranes using 0.2% Triton X100 (AmericanBio, Natick, MA, USA) in PBS for 2-3 minutes and blocking with 2% bovine serum albumin (BSA) in PBS (Sigma-Aldrich, USA) for 1 hour. The next day, cells first were washed in 1% Tween20 (AmericanBio, Natick, MA, USA) in PBS for 1-2 min, 0.2% TritonX100 in PBS twice for 2-3 min (2X) and finally with 0.1% BSA in PBS for 5 min and subsequently were incubated with 1:500 dilutions of secondary anti-rabbit DyLight®488 (green; Vector Labs, USA), for 1 hour and 30 min, at room temperature, washed and mounted using Prolong Gold Mountant with diamidino-phenylindole (ProLong® Diamond Antifade Mountant with DAPI; Life Technologies, Thermo Scientific, MA, USA) for nuclear staining and mounting of cells (blue). The slides were examined using a Zeiss Confocal microscope and images were captured and analyzed using Zen imaging software from Carl Zeiss, microscopy (Germany).

***miRNA analysis***

We performed miRNA analysis in order to determine the expression levels of miR-21, -155, -192, -34a, -375, -451a, previously characterized in laryngopharyngeal cancer [12-17] and/or acidic bile related miRNA markers [10], in normal human hypopopharyngeal cells exposed to acidic bile and corresponding controls with or without pharmacologic inhibitor BAY 11-7082 [11]. Total RNA was isolated from human hypopharyngeal primary cells (HHPC) and human hypopharyngeal cells (HHK) exposed to acidic bile (pH 4.0), neutral bile (pH 7.0), acid (pH 4.0) and neutral control (pH 7.0) fluids, with or without BAY 11-7082, using RNeasy mini kit (Qiagen®, KY, USA). RNA quality and concentration ratios were determined by absorption at 260 nm were determined using a NanoDrop^TM^ 1000 spectrophotometer (Thermo Scientific). We subsequently performed reverse transcription synthesis of miRNAs from total RNA, using miScript II RT kit (Qiagen®, KY, USA), according to the manufacturer’s instructions. In order to quantify specific miRNA markers, we performed real-time qPCR analysis (Bio-Rad real-time thermal cycler CFX96TM), using specific primers for target-miRNAs of human genome (miScript Primer Assays, Qiagen®, KY, USA) and normalization control small RNA [snRNA RNU6B (RNU6-2), Qiagen®, KY, USA] (Supplementary Table S1), as well as miScript SYBR Green PCR Kit (Qiagen®, KY, USA). Real time qPCR assays were performed in 96 well-plates and each sample was assayed in triplicate. We used CFX96TM software (Bio Rad, CA, USA) to analyze our data. We estimated relative expression levels (target miRNA/RNU6B) for each specific miRNA marker, in each experimental and control group treated with or without NF-*κ*B inhibitor (CFX96TM software; Bio Rad, CA, USA). (Data were obtained from three independent experiments.)

## *Quantitative real time PCR*

We performed quantitative real time polymerase chain reaction (qPCR) analysis in order to quantify the transcriptional levels of RELA (p65), TNF-α, L-1β, IL-6, and STAT3, in HHPC exposed to GDF and corresponding controls, with or without BAY 11-7082, as previously described [8,33]. We isolated total RNA (RNeasy mini kit; Qiagen Inc., CA, USA) from HHPC and we determined RNA quality and concentration by absorption ratios at 260/280 nm (>2.0) and 260 nm, respectively (NanoDrop^TM^ 1000 spectrophotometer; Thermo Fisher Scientific, Waltham, MA). Subsequently, we performed reverse transcription (iScript cDNA synthesis kit; Bio-Rad) and real time qPCR analysis (Bio-Rad real time thermal cycler CFX96TM; Bio-Rad), using specific primers for target genes and reference housekeeping gene, human glyceraldehyde 3-phosphate dehydrogenase (*h*GAPDH) (Supplementary Table S2), (QuantiTect Primers Assays; Qiagen), and iQ^TM^ SYBR Green Supermix (Bio-Rad). We performed assays in 96-well plates, in triplicate for each sample, and data were analyzed by CFX96^TM^ software. Relative mRNA expression levels were estimated for each target gene relative to reference gene (ΔΔ*C*t). (Data were obtained from three independent experiments.)

## *Cell viability assay*

We performed a cell viability assay, using Cell Titer-Glo® Luminescent Cell Viability Assay (Promega). The cells were seeded at a density 5,000 and 10,000 cells/well for HHK and HHPC, respectively, in 24-well plates. The next day the cells underwent a single exposure with experimental (acidic bile at pH 4.0, neutral bile at pH 7.0, with or without BAY 11-7082) and control fluid (acid alone at pH 4.0, neutral control at pH 7.0, with or without BAY 110-782) for 1 hour. At the end of the treatment, we removed the media and we replaced them with serum free basal media (Human Hypopharyngeal Normal Cell Culture Media Serum Free, for HHPC cells, from Celprogen Inc. CA, USA, and KGM-2 SF, for HHK cells, from Gibco®, NY, USA). Cells were cultured at 37°C in humidified air and 5% CO2 for 7 days. Subsequently, we used a luminometer to measure the luminescence. All values were normalized to mean value of untreated controls. We determined the cell viability comparing the mean values of cells exposed with NF-*κ*B inhibitor against the mean value for cells did not exposed with inhibitor, for each experimental and control group. Supplementary Figure S3 was obtained from three independent experiments. Statistically significant difference of cell viability was determined using paired-test and p value <0.05 (Graph Pad Prism 6.0).

**Supplementary Table S1:** Targets for human mature miRNA and RNU6-2 small RNA control, analyzed by real time qPCR, in human hypopharyngeal primary cells exposed to gastroduodenal fluid (pH 4.0 and 7.0) and corresponding controls, with and without BAY 11-7082.

| **miRNA (human)** | **Target mature miRNA** (Sanger Accession) |
| --- | --- |
| miR-21 | [hsa-miR-21-5p](http://www.mirbase.org/cgi-bin/mature.pl?mature_acc=MIMAT0000076), MIMAT0000076 |
| miR-192 | [hsa-miR-192-5p](http://www.mirbase.org/cgi-bin/mature.pl?mature_acc=MIMAT0000222), MIMAT0000222 |
| miR-155 | [hsa-miR-155-5p](http://www.mirbase.org/cgi-bin/mature.pl?mature_acc=MIMAT0000646), MIMAT0000646 |
| miR-375 | [hsa-miR-375](http://www.mirbase.org/cgi-bin/mature.pl?mature_acc=MIMAT0000728), MIMAT0000728 |
| miR-34a | [hsa-miR-34a-5p](http://www.mirbase.org/cgi-bin/mature.pl?mature_acc=MIMAT0000255), MIMAT0000255 |
| miR-451a | [hsa-miR-451a](http://www.mirbase.org/cgi-bin/mature.pl?mature_acc=MIMAT0001631), MIMAT0001631 |
| **Small RNA** | **Control** |
| RNU6-2 (RNU6-6P RNA, U6 small nuclear 6, pseudogene) | Hs_RNU6-2_11 |

**Supplementary Table S2:** Human genes analyzed by real-time qPCR, in normal human hypopharyngeal cells.

| **Gene** | **Detected transcripts** | **Amplicon length (bp)** |
| --- | --- | --- |
| ***h*GAPDH** | NM_001256799, NM_002046 | 95 |
| **RELA** | NM_001145138, NM_001243984-5, NM_021975 | 107 |
| **TNF** | NM_000594 | 98 |
| **STAT3** | NM_003150,  NM_139276 | 95 |
| **IL-1β** | NM_000576, XM_006712496 | 117 |
| **IL-6** | NM_000600 XM_005249745 | 107 |

***S*upplementary Figure S1: BAY 11-7082 demonstrates more intense changes of cancer-related miRNA markers in acidic bile (pH 4.0) relative to neutral bile (pH 7.0) treated normal human hypopharyngeal cells.** Graphs depict significant changes of **(A)** “oncomirs” and “tumor suppressor” miRNAs in acidic bile vs neutral bile treated **(a)** HHPC and **(b)** HHK with versus without NF-κB inhibitor (BAY 11-7082). **B.** Table presents acidic bile and neutral bile induced miRNA ratios (with/without BAY 11-7082) in treated normal human hypopharyngeal cells (HHPC and HHK) and statistical analysis results from their comparison (p values by t-test; mean ±SD; GraphPad Prism 6.0). (Data were obtained from three independent experiments.)


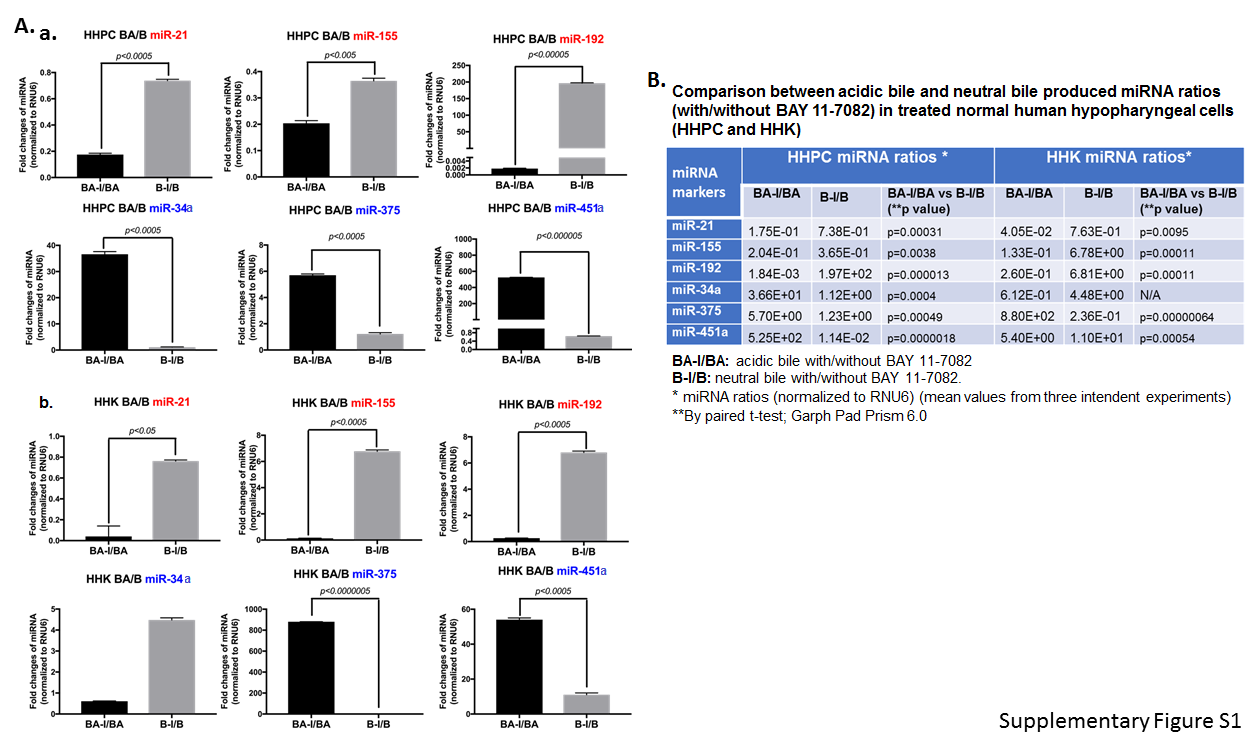


.

**Supplementary Figure S2: Correlations among BAY 11-7082-induced oncogenic miRNA expression ratios (with/without inhibitor) in treated normal human hypopharyngeal cells.** Graphs depict a strongly inverted correlation between BAY 11-7082-induced expression changes of **(a)** miR-21 and miR-375 in normal human hypopharyngeal cells (both HHK and HHPC), **(b)** miR-155 and miR-451a in HHPC, **(c)** miR-192 and miR-451a in HHPC, **(d)** miR-155 and miR-375 in HHK and **(e)** miR-192 and miR-451a in HHK (by Pearson, p value <0.05).


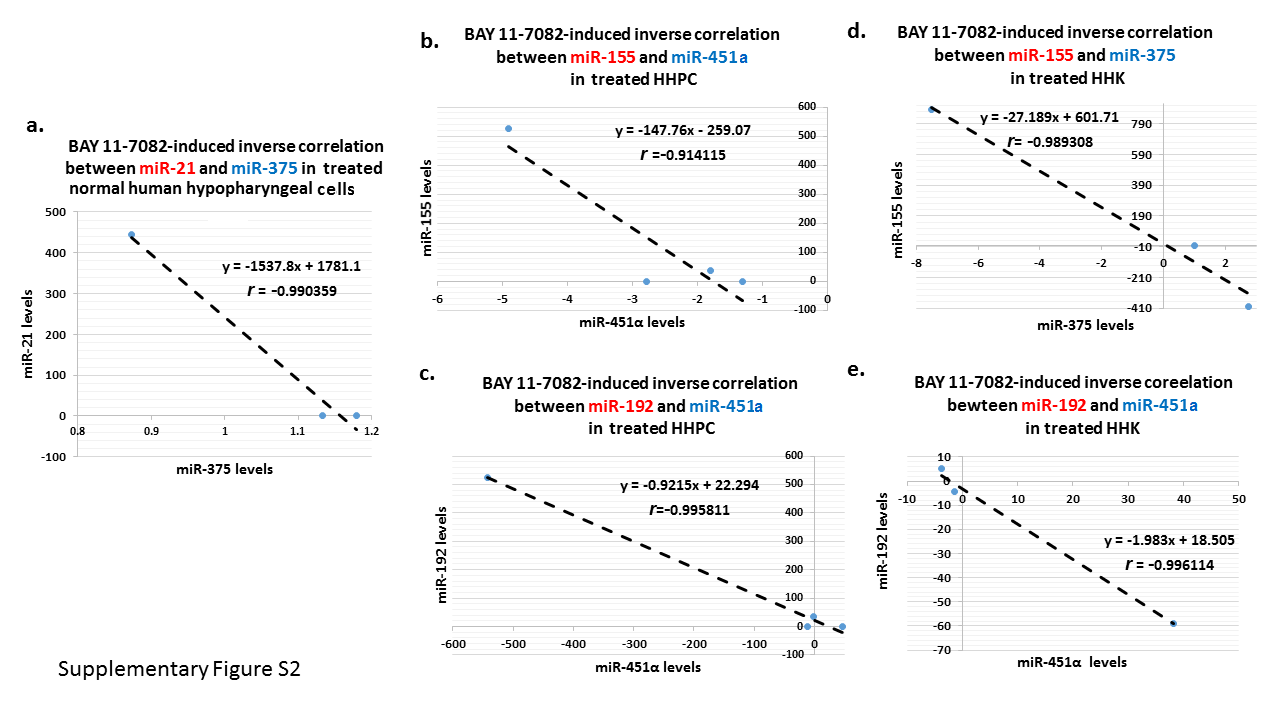


**Supplementary Figure S3. Cell viability assay demonstrates that 20 μM of BAY 11-7082 reduces the viable acidic bile treated normal human hypopharyngeal cells (HHPC and HHK) (A)** Cell viability in HHPC exposed **(a)** to neutral bile or neutral control **(b)** acidic bile or acid alone, with and without BAY 11-7082. **(B)** Cell viability in HHK exposed **(a)** to neutral bile or neutral control **(b)** acidic bile or acid alone, with and without BAY 11-7082. **(C)** DMSO had no negative effects on cell viability of **(a)** HHPC and **(b)** HHK treated cells, indicated by similar percentages of viable cells compared to controls.


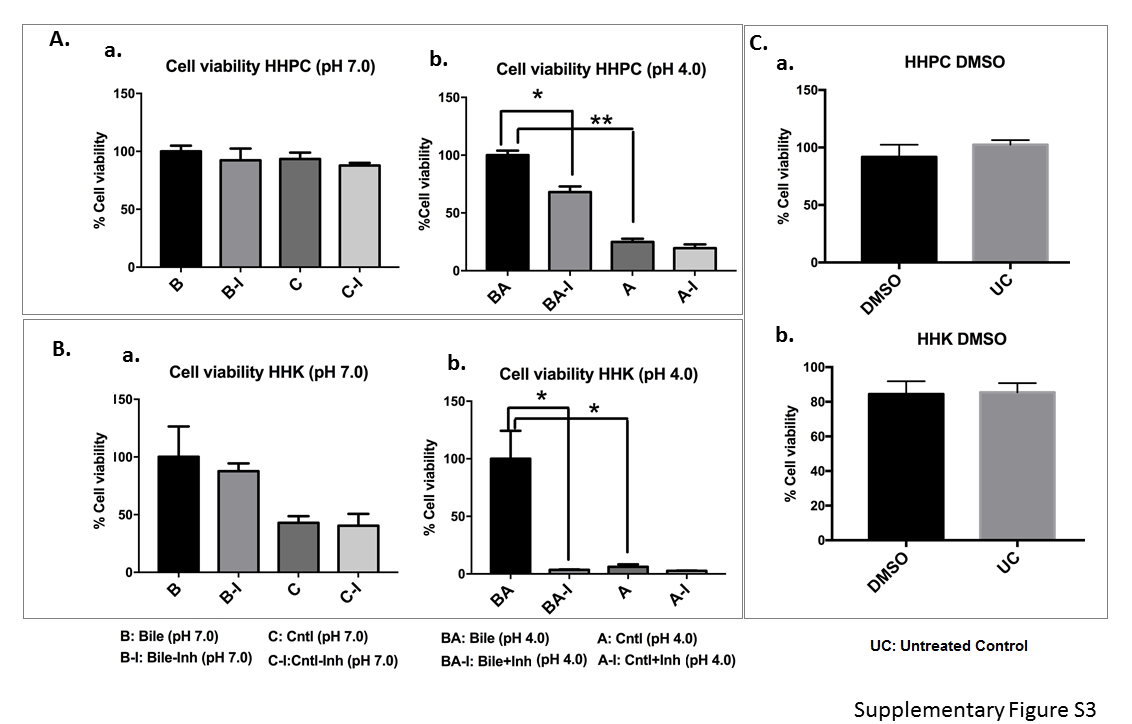


**Supplementary Table S3:** Relative expression ratios of the analyzed, by real time qPCR, genes in human hypopharyngeal primary cells exposed to gastroduodenal fluid (pH 4.0 and 7.0) and corresponding controls, with and without BAY 11-7082.

|  | **HHPC** | | | | |
| --- | --- | --- | --- | --- | --- |
| **Target gene/**  ***h*GAPDH (*ΔΔ^CT^*)** | **pH 7.0** | | **pH 4.0** | | |
|  | **C-I*/C** | **B-I/B** | | **A-I/A** | **BA-I/BA** |
| **RELA (p65)** | 3.93E-01 | 8.53E-01 | | 1.07E-01 | 1.96E-03 |
| **STAT3** | 5.13E-01 | 4.41E-01 | | 2.30E-01 | 3.78E-04 |
| **TNF-α** | 1.00E-01 | 1.67E+00 | | 1.09E+00 | 1.33E-02 |
| **IL-1β** | 1.15E+00 | 1.09E+00 | | 4.48E-02 | 2.06E-02 |
| **IL-6** | 3.75E-01 | 2.65E+00 | | 5.25E-01 | 4.67E-02 |

*I: Inhibitor (BAY 11-7082)
